# The antimicrobial resistance landscape of slaughterhouses in western Kenya: A microbiological case study

**DOI:** 10.1016/j.onehlt.2024.100899

**Published:** 2024-09-19

**Authors:** Katie A. Hamilton, Sam M. Njoroge, Kelvin Momanyi, Maurice K. Murungi, Christian O. Odinga, Nicholas Bor, Allan Ogendo, Josiah Odaba, Joseph G. Ogola, Eric M. Fèvre, Laura C. Falzon

**Affiliations:** a Institute of Infection, Veterinary and Ecological Sciences, University of Liverpool, Liverpool L69 3BX, United Kingdom; b International Livestock Research Institute, P.O. Box 30709-00100, Nairobi, Kenya; c Kenya Medical Research Institute, P.O. Box 54840-00200, Nairobi, Kenya; d Directorate of Veterinary Services, County Government of Busia, P.O. Box 261-50400, Busia, Kenya; e Directorate of Veterinary Services, County Government of Bungoma, P.O. Box 135-50200, Bungoma, Kenya

**Keywords:** Microbiological assessment, Multi drug resistance, Food value chain

## Abstract

Slaughterhouses may be hotspots for the transmission of antimicrobial resistant (AMR) pathogens. To obtain information on the AMR landscape in Kenyan slaughterhouses, we collected swabs of the environment, animal carcasses, and workers. Bacterial isolates were identified in 101/193 (52.3 %) samples, and most showed resistance to streptomycin (68.7 %), ampicillin (48.7 %), and tetracycline (42.5 %). Multi drug resistance was exhibited by 35/80 isolates (43.8 %; 95 % CI: 33.2–54.9 %), while Extended Spectrum Beta Lactamase was expressed in 5/80 isolates (6.3 %; 95 % CI: 2.6–14.3 %). These findings illustrate the presence of resistant bacteria throughout the slaughterhouse environment, posing a risk to workers and meat consumers and highlighting the need for an integrated surveillance system along the food chain.

## Highlights

• We identified a high frequency of drug resistant bacterial pathogens in the slaughterhouse environment.
• Presence of resistant bacterial pathogens in carcass and meat box samples demonstrates risk in the food value chain.
• Trend of higher resistance in pig slaughterhouses merits further investigation.

## 1 Introduction

Antimicrobial resistance (AMR) arises as a pathogen evolves to no longer respond to antimicrobials that were previously effective [1]. Antimicrobial resistance is a serious public health concern across the globe [2] with 1.27 million deaths linked to AMR infections reported in 2019, most of those in Low- and Middle-Income Countries (LMICs) [3]. Over the last decade, increasing levels of resistance to clinically relevant antibiotics, including carbapenems and colistin, which are considered antibiotics of last resort, have been reported in both human and animal populations [4].

The health of humans, animals and the environment are dependent on each other, an intrinsic link termed One Health [5]. Antimicrobial resistant pathogens can spread between the environment, human, and animal communities [6], and the health of an animal can influence human health both directly, and indirectly through the consumption of infected livestock products and contamination of shared environments [5]. Antimicrobial drugs are easily accessible to livestock farmers in many countries, without the need for a prescription, making it difficult to monitor antimicrobial use in food-producing animals [7]. The ease of accessibility of antimicrobials is a common problem in many LMICs [8], as was illustrated by Muloi et al. [9] in Kenya.

In western Kenya, previous studies have illustrated the prevalence of zoonotic diseases in slaughterhouses and the risk of disease due to exposures in these working environments [[10], [11], [12], [13], [14]]. However, there are limited studies focussed on the risk of transmission of resistant bacteria [15] and the repercussions for public health and food safety in the region.

The aim of this study was to understand whether drug resistant bacteria are circulating within the slaughterhouse working environment in western Kenya, using *Escherichia coli* as an exemplar pathogen. *E. coli* has been classified as a priority pathogen by the World Health Organization (WHO) due to its worldwide antibiotic resistance [2]. This baseline information on the AMR situation in the slaughterhouse context will inform future research and surveillance work.

## 2 Methods

### 2.1 Study area

This study was embedded within a larger surveillance study in Busia, Bungoma and Kakamega Counties in western Kenya [16], which involved sampling in twelve sentinel sites across the three Counties. Each site comprised of a livestock market, one or two slaughterhouses, and a hospital, and was visited every four weeks over a two-year period. During each visit, up to ten animals at both livestock markets and slaughterhouses, and up to ten patients at hospitals, were sampled. All samples were taken to the field laboratory in Busia where they were processed and tested for several zoonotic diseases.

The work described here focused on the slaughterhouses in these twelve sites and was conducted between February and May 2019. Specifically, 13 slaughterhouses (9 ruminant and 4 pig) in 11 sentinel sites (4 in Busia County, 3 in Bungoma County, and 4 in Kakamega County) were included. One of the ruminant slaughterhouses was classified as Category B [medium], with an expected animal throughput ranging between 6 and 39 cattle and 16–24 small ruminants. The other 8 ruminant slaughterhouses were Category C slaughter slabs, with an expected throughput not exceeding 5 cattle or 15 small ruminants [17]. All the pig slaughterhouses were informal slaughter slabs. These slabs are customarily located in rural areas and, having little or no facilities [18] do not meet the minimal requirements stipulated in the Meat Control Regulations [17]. The number of workers at each slaughterhouse ranged between 5 and 24; these included the person(s) who carried out the slaughter, flayers who dressed the carcass, gut cleaners, and site cleaners. For this work, each slaughterhouse was visited once, and this visit coincided with the routine longitudinal visits of the larger surveillance study.

### 2.2 Description of slaughterhouse process

The slaughterhouses included in this study (Category B, C, and informal slabs) are representative of the slaughterhouses in the study area and represent a large proportion of slaughter facilities serving the majority of Kenya's population [14]. Except for the Category B slaughterhouse which had a mechanical chute for restraining animals and used a stunning gun prior to slaughter, in all the other slaughterhouses the animals were manually restrained and slaughtered. Once the animal was dead, the flayers would start eviscerating and dressing the carcass. This was initially done on the floor, after which the carcass would be hoisted using chains or ropes, and the carcass dressing finalised. In some slaughterhouses, the dressed carcass would be placed on a meat slab for meat inspection, while in others the carcass remained hoisted. Following inspection, pieces of or the whole carcass would be placed in a meat box and transported to the butchery. Throughout the slaughter process, cleaners would collect and clean the viscera and pour water on the surfaces to wash out blood and other effluents. Some slaughterhouses had boreholes or wells for water, while others brought water in jerry cans or from a nearby river. Our microbiological assessment was therefore designed to sample the working environment, as well as the actors involved in the process and the equipment used.

### 2.3 Slaughterhouse sampling

**Fig. 1:**
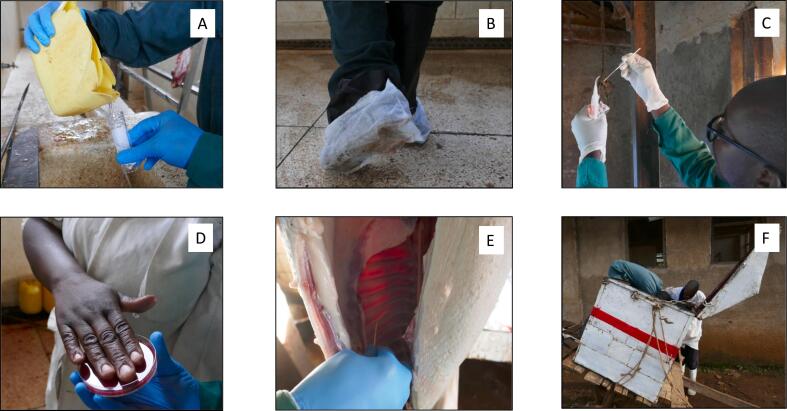
Microbiological assessments in slaughterhouses in western Kenya. Samples included water samples (A), floor samples – boot socks (B), swabs of ropes/chains (C), hand swabs of slaughterhouse workers (D), swabs of an animal carcass (E), meat box (F).

During the site visit, a microbiological assessment of the working environment, workers, animal carcasses, and meat transport system in each slaughterhouse was conducted (Fig. 1). Water samples were collected directly from the storage container of fresh water or tap into a sterile pot. The slaughterhouse floor was sampled by walking across the floor using boot covers dipped in saline solution; this was done before animals were slaughtered or any water was poured on the floor for cleaning. Transwabs® with liquid Amies (Medical Wire and Equipment, UK) were used to swab both the knives used for slaughter and flaying the carcass, and the ropes or chains used to hoist the carcass. Both hands of the flayer and/or gut cleaner were sampled, either using swabs or by taking hand smears directly on MacConkey agar (Oxoid, UK) plates. Equipment and hand samples were taken before or during the slaughter process. Following slaughter, one dressed carcass was swabbed in three points (shoulder, rump and inside) with a gauze soaked in Buffered Peptone Water (BPW); a metal template measuring 10 by 10 cm^2^ was used to determine the surface area to be swabbed. The inside of a meat box (standardized metal boxes used to transport meat from slaughterhouses to butcheries), including all four corners, was swabbed before any meat had been placed inside.

The boot covers were placed in a dry ZipLoc® bag, while the gauzes were placed in BPW. Samples were safely stored and maintained at room temperature (24–28 °C) during transport; the driving distance between the slaughterhouses and the field laboratory in Busia County ranged between 30 and 120 min. Duplicate samples were collected when possible, and the same investigators collected the samples in all slaughterhouses to limit sampling error.

### 2.4 Laboratory analysis

Once at the laboratory, the samples were enriched in Luria Bertani broth (Sigma Aldrich) and left overnight at 37 °C. A 5 μL inoculum was then plated on both Eosin Methylene Blue agar and MacConkey agar (Oxoid UK) and incubated overnight at 37 °C. Presumptive lactose fermenter colonies were picked for biochemical analysis, and those identified as *E. coli* were subjected to antimicrobial susceptibility testing using the Kirby Bauer disk diffusion method (Oxoid, UK). Antibiotic discs were dispersed onto Müller Hinton agar containing an even lawn culture of 0.5 McFarland in 0.85 % saline and incubated overnight at 37 °C. Clinical and Laboratory Standards Institute interpretive criteria for Enterobacteriaceae were used to determine breakpoints for classifying isolates as either susceptible (‘susceptible’ or ‘intermediate’) or non-susceptible (‘resistant’) [19]. The isolates that grew were tested against a range of 14 antibiotics: ampicillin (10 μg/mL), amoxicillin-clavulanic acid (30 μg/mL), aztreonam (30 μg/mL), cefepime (30 μg/mL), cefotaxime (30 μg/mL), ceftazidime (30 μg/mL), chloramphenicol (30 μg/mL), ciprofloxacin (5 μg/mL), gentamicin (10 μg/mL), nalidixic acid (30 μg/mL), streptomycin (25 μg/mL), sulfamethoxazole (30 μg/mL), tetracycline (30 μg/mL), and trimethoprim (2.5 μg/mL). Standardized protocols were used, in which antibiotic discs were dispensed on to bacteria-containing agar plates and incubated for a maximum of 18 h at 37 °C. Any suspected Extended Spectrum Beta Lactamase (ESBL) producing isolates were identified phenotypically and tested using the double disc synergy test [20,21]. Briefly, the formation of a ghost inhibition zone between amoxicillin-clavulanic acid and third generation cephalosporins was predictive of ESBL producing *E. coli*, in addition to resistance to the other classes of antibiotics, namely (fluoro)quinolone, aminoglycosides and/or cephamycin. As a susceptible quality control, *E. coli* ATCC 25922 was used for all the susceptibility tests.

### 2.5 Data analysis

Laboratory data were entered into a Microsoft Excel (Microsoft, Redmond, WA, USA) spreadsheet and cleaned. Isolates resistant to three or more antimicrobial classes were defined as Multi Drug Resistant (MDR). Statistical analyses were carried out using Stata Statistical Software: Release 18 (College Station, TX: StataCorp LP). Categorical variables were summarized, and the frequency and 95 % Confidence Intervals (CI) of MDR and ESBL was determined. A Fisher's exact test was used to check for statistical differences (*p*-value <0.05) in the frequency of isolates, MDR, and ESBL in pig and ruminant slaughterhouses.

### 2.6 Ethical approval

All procedures were in accordance with the ethical standards of the Helsinki Declaration of the World Medical Association. The design of the study was approved by the Institutional Animal Care and Use Committee (IACUC Reference No. 2017–04) and the Institutional Research Ethics Committee (IREC Reference No. 2015–10/3) at the International Livestock Research Institute, review bodies accredited by the Kenyan National Commission for Science, Technology and Innovation (NACOSTI), and approved by the Federal Wide Assurance for the Protection of Human Subjects in the United States of America. Approval to conduct the work was also obtained from the Kenya Department of Veterinary Services and the relevant offices at devolved government level.

## 3 Results

We collected 193 samples from 13 (9 ruminant and 4 pig) slaughterhouses in 11 sentinel sites. These included 57 (29.5 %), 65 (33.7 %), and 71 (36.8 %) samples from slaughterhouses in Busia, Bungoma, and Kakamega, respectively. There were 8 sample types: carcass (*n* = 40; 20.7 %), flaying and slaughter knives (n = 40; 20.7 %), flayers and cleaners' hands (*n* = 24; 12.4 %), hoisting rope or chains (*n* = 22; 11.4 %), floor (n = 22; 11.4 %), water (n = 22; 11.4 %), meat box (*n* = 19; 9.8 %), and meat slab (n = 4; 2.1 %). Of the 193 samples, 53 (27.5 %) and 140 (72.5 %) were collected from a pig and ruminant slaughterhouse, respectively.

**Fig. 2:**
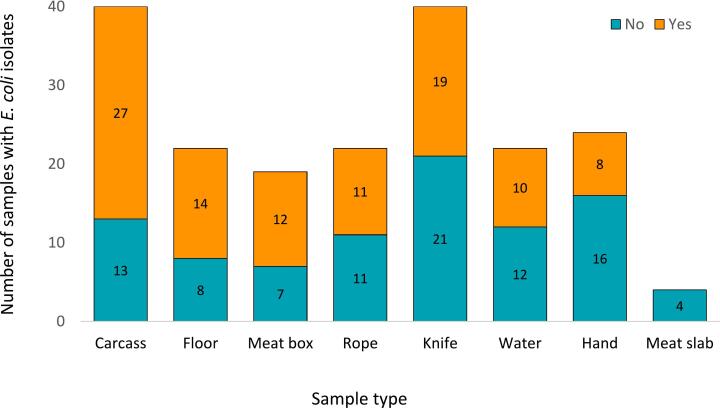
Number of isolates identified in different sample types collected from thirteen slaughterhouses in western Kenya.

Bacterial isolates were identified in 101/193 (52.3 %) samples; these 101 samples were collected from the 11 sentinel sites in Busia (*n* = 33; 32.7 %), Bungoma (*n* = 28; 27.7 %), and Kakamega (*n* = 40; 39.6 %), and included 7 sample types. The highest proportion of isolates were identified in the carcass (27/40; 67.5 %), floor (14/22; 63.6 %), and meat box (12/19; 63.2 %) samples (Fig. 2). There was no statistically significant difference in the proportion of isolates from samples collected in pig (25/53; 47.2 %) and ruminant (76/140; 54.3 %) slaughterhouses (*p* = 0.42).

**Fig. 3:**
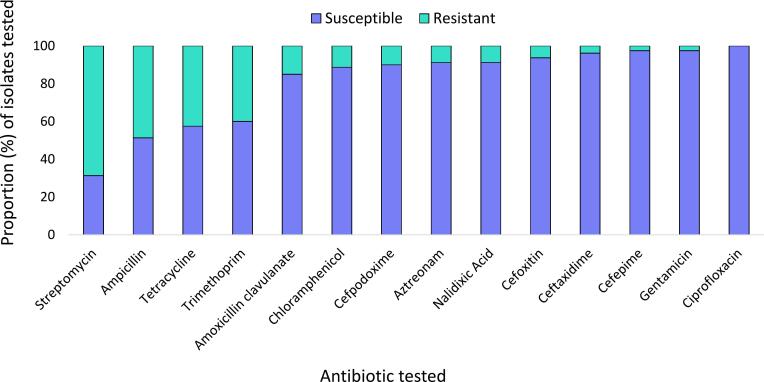
Proportion of isolates showing resistance to the fourteen antibiotic drugs assessed with Antimicrobial Susceptibility Tests.

Antimicrobial susceptibility tests were undertaken on 80 of the 101 isolates; the remaining isolates were not used either because there was insufficient material to conduct ASTs (*n* = 12), or because they grew on duplicate plates containing cefotaxime (*n* = 9) and were therefore excluded to avoid biasing the results. The 80 isolates were from carcass (*n* = 25), knife (*n* = 14), meat box (n = 12), floor (n = 9), rope (n = 9), water (*n* = 7), and hand (*n* = 4) samples. The highest frequencies of resistance were observed towards streptomycin (68.7 %), ampicillin (48.7 %), and tetracycline (42.5 %), while none of the isolates showed resistance to ciprofloxacin (Fig. 3).

**Fig. 4:**
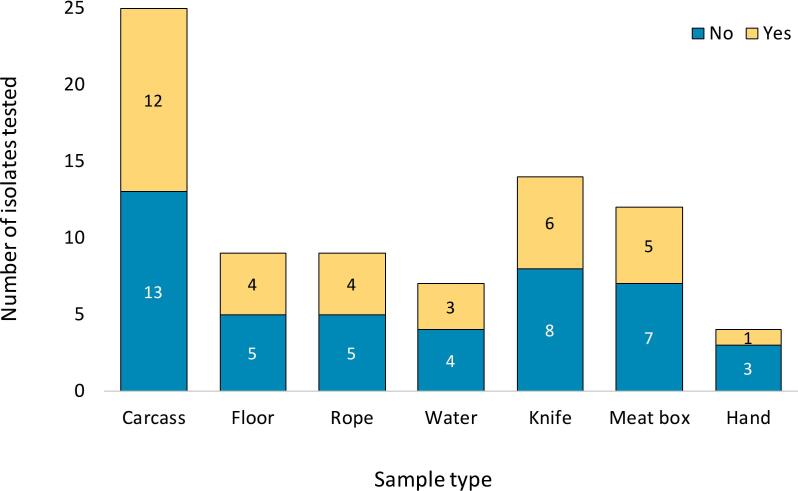
Number of isolates that exhibited Multi Drug Resistance in each sample type.

Each isolate was resistant to a median of 2 drugs (ranging from 0 to 10). Multi drug resistance was exhibited by 35/80 isolates (43.8 %; 95 % CI: 33.2–54.9 %). These MDR isolates were found in samples from all 11 sentinel sites in Busia (9/26; 34.6 %), Bungoma (10/27; 37.0 %), and Kakamega (16/27; 59.3 %). Carcass (12/25; 48.0 %), floor (4/9; 44.4 %) and rope (4/9; 44.4 %) samples had the highest proportion of MDR isolates (Fig. 4). The difference in the proportion of MDR isolates present in pig (11/20; 55.0 %) and ruminant (24/60; 40 %) slaughterhouse samples was not statistically significant (*p* = 0.30).

Extended Spectrum Beta Lactamase was expressed by 5 of the 80 isolates (6.3 %; 95 % CI: 2.6–14.3 %). These 5 ESBL positive samples were collected in 3 of the 11 sites and comprised 3 sample types. Specifically, 3 isolates were identified in a rope, floor, and meat box sample, all taken from the same pig slab in Busia; the other 2 isolates were both identified in meat box samples, one taken from a pig slab in Kakamega and the other taken from a ruminant slaughterhouse in Bungoma. The proportion of ESBL positive samples identified in pig (4/20; 20.0 %) and ruminant (1/60; 1.7 %) slaughterhouse samples was statistically different (*p* = 0.01).

## 4 Discussion

This case study aimed to understand the frequency and distribution of antimicrobial resistant pathogens within the slaughterhouse setting. Results showed a high frequency of resistant pathogens in both carcass and environmental samples, leading to potential contamination of the surroundings and along the food chain. These findings highlight the need to better understand the source and direction of AMR transmission, and to determine critical points for intervention and surveillance activities.

The drugs towards which the highest resistance was shown were streptomycin, ampicillin, and tetracycline. This was not surprising as they correspond to the antibiotics most frequently used, as mentioned in stakeholder discussions held in parallel with the work reported here [15], and as reported by farmers and pharmacists in another study conducted in the same area [22]. Other studies that looked at antimicrobial resistant pathogens in raw pork and poultry in Kenya detected a high rate of resistance frequencies against tetracycline and ampicillin (39 % and 35 %, respectively), confirming a heavy use of these antibiotics in the poultry and pork farming industries [23]. This is further highlighted in cattle production systems in Kenya, with tetracycline and streptomycin being listed amongst the most frequently administered antibiotics [24].

The rationale for these patterns of AMR transmission is related to the intensive farming methods, lack of space, reducing disease transmission and lack of biosecurity within farms The findings are also in line with other studies conducted within Nairobi, Kenya, where more than half of the *E.coli* isolates causing diarrhoea were resistant to ampicillin, tetracycline and streptomycin [25,26]. Other studies in Kenya have shown the ease with which antibiotics can be freely obtained and administered with no medical supervision [27] and no guidance on how to use antibiotics [24]. On the other hand, none of the isolates showed resistance to ciprofloxacin, an antibiotic classed by the WHO as an essential medicine [28].

Almost half of the isolates tested (43.8 %) exhibited MDR, and the highest proportion of MDR isolates were found in carcass, floor, and rope samples. A similar study performed in Kenyan bovine slaughterhouses and looking at antimicrobial resistance of enterococci.

identified 14.9 % of isolates which were resistant to 3 or more antibiotics, while 30 % of the isolates exhibited resistance to at least one antibiotic [29]. The carcass isolates could be due to internal contamination during evisceration, or external contamination during processing and handling, highlighting the importance of training on meat handling. Education was indeed one of the recurring themes highlighted by the slaughterhouse workers in the accompanying stakeholder discussions [15]. The majority of slaughterhouse workers within Kenya have not been educated in slaughter hygiene and meat safety [29]. The floor and rope samples represent the environment and what is circulating throughout the system, and the presence of resistant isolates could be exacerbated by the lack of biosecurity and reliable water sources in some of the slaughterhouses, also highlighted in the stakeholder discussions [15], and lack of chemical disinfection of these sites. [30,31].

Only 5 of the 80 isolates expressed ESBL. These were primarily found in samples collected from the meat boxes. These are metal containers with a lid used to transport raw meat to the butchers – Fig. 1 (f), a legal requirement in Kenya to transport raw meat. These results highlight the risk of resistant bacteria entering the food chain through food chain infrastructure. Furthermore, the number of isolates from samples collected in pig slaughterhouses was statistically higher than that from ruminant slaughterhouse samples. While the sample size is small and statistical significance should be interpreted with caution, this trend merits further investigation given that most pig slabs in western Kenya are informal with limited infrastructure and biosecurity measures.

Slaughterhouses in western Kenya are often embedded within the community, at the nexus of human, animal, and environmental health. They are often situated within the context of a village trading centre, and as such are very much a part of the broader community. Workers who provide informal employment in the slaughterhouse are drawn from nearby farming communities [32]. Traders and butchers also play a pivotal role in the meat value chain network [32,33]. Traders purchase animals either directly from the farmer or from livestock markets, bring the livestock to the slaughterhouse where they pay for slaughter services, and then transport the carcass to a retail outlet; the meat is sold to restaurants or directly to consumers [34,35]. Some butchers also purchase their own live animals, bring them directly to slaughter, and then have the carcases delivered to their own butchery [33].

While these frequent interactions between the slaughterhouse, workforce, and surrounding community increase the risk of disease transmission, AMR spread, and pathogen spill over, slaughterhouses also provide an important entry point for interventions which can then have ripple effects on public health, animal health and welfare, and food safety.

A limitation of this study was the lack of sequence data which would have given us a more insightful picture of the true nature of carriage of AMR *E. coli*. Due to the limited sample size, we collapsed certain categories (e.g., flayers and cleaners' hands; slaughter and flaying knife), and we were also restricted in our ability to investigate statistically significant differences between sample types. Future work could include more slaughterhouses or sample the same sites more frequently to shed light on possible differences both between and within the slaughterhouses. Furthermore, future research could include the poultry sector (where slaughter is usually not done in centralised slaughter facilities) and consider following the meat from the slaughterhouse via the butcher to the consumer to better understand the risk along the food chain, as recently undertaken for Salmonella in the same setting [36].

This case study provides baseline information on the frequency and distribution of resistant pathogens in the slaughterhouse setting. Findings highlight the importance of training on food handling and hygiene measures to reduce carcass contamination, as well as the need to invest in better infrastructure and biosecurity measures to mitigate the spread of resistant pathogens along the food chain and in the surrounding environment.

## Role of the funding source

This work was supported by the 10.13039/501100000268UK Biotechnology and Biological Sciences Research Council Impact Acceleration Award (BB/S506746/1) to the 10.13039/501100000836University of Liverpool, and by the 10.13039/501100000836University of Liverpool Knowledge Exchange and Impact and Public Engagement Voucher Schemes. The study was also part of the Zoonoses in Livestock in Kenya (ZooLinK) project, which was supported by the 10.13039/501100000268Biotechnology and Biological Sciences Research Council, the 10.13039/501100000278Department for International Development, the Economic & Social Research Council, the 10.13039/501100000265Medical Research Council, the 10.13039/501100000270Natural Environment Research Council, and the Defence Science & Technology Laboratory, under the Zoonoses and Emerging Livestock Systems (ZELS) programme, grant reference BB/L019019/1. We also received support from the CGIAR One Health initiative “Protecting Human Health through a One Health Approach”, which was supported by contributors to the CGIAR Trust Fund (https://www.cgiar.org.funders/).

## CRediT authorship contribution statement

**Katie A. Hamilton:** Writing – review & editing, Writing – original draft, Methodology, Investigation, Funding acquisition, Formal analysis, Data curation, Conceptualization. **Sam M. Njoroge:** Writing – review & editing, Methodology, Formal analysis, Data curation. **Kelvin Momanyi:** Writing – review & editing, Methodology, Investigation. **Maurice K. Murungi:** Writing – review & editing, Methodology, Investigation. **Christian O. Odinga:** Writing – review & editing, Methodology, Investigation. **Nicholas Bor:** Writing – review & editing, Methodology, Investigation. **Allan Ogendo:** Writing – review & editing, Methodology, Investigation. **Josiah Odaba:** Writing – review & editing, Methodology, Investigation. **Joseph G. Ogola:** Writing – review & editing, Methodology, Investigation. **Eric M. Fèvre:** Writing – review & editing, Methodology, Investigation, Funding acquisition, Data curation, Conceptualization. **Laura C. Falzon:** Writing – review & editing, Writing – original draft, Methodology, Investigation, Funding acquisition, Formal analysis, Data curation, Conceptualization.

## Declaration of competing interest

None.

## Data Availability

The datasets generated and/or analysed during the current study are available in the Liverpool DataCat: The Research Data Catalogue at https://doi.org/10.17638/datacat.liverpool.ac.uk/2665.
